# Editorial: Machine learning and AI-driven insights into microbial pathogenesis and drug resistance

**DOI:** 10.3389/fcimb.2026.1980955

**Published:** 2026-09-10

**Authors:** Zhi-Kai Yang, Yanming Zhang

**Affiliations:** 1 Key Laboratory of Biological Targeting Diagnosis, Therapy and Rehabilitation of Guangdong Higher Education Institutes, The Fifth Affiliated Hospital of Guangzhou Medical University, Guangzhou, China; 2 SinoGenoMax Co., Ltd./Chinese National Human Genome Center, Beijing, China

**Keywords:** anti-microbial resistance, clinical prediction models, explainable artificial intelligence, host–pathogen interactions, machine learning

To survive in drug- or antibiotic-exposed environments, microbes must undergo rapid adaptive evolution. Therefore, the emergence and continuous strengthening of antibiotic resistance, along with its global spread, pose a significant threat to modern medicine and human health (World Health Organization, 2025). Driven by the rapid advancement of high-throughput omics and sequencing technologies, it has become feasible to efficiently generate large-scale disease-associated phenotypic and genomic datasets, which provides a robust foundational framework for in-depth mechanistic investigations of pathogenesis and antimicrobial tolerance. However, due to the complexity and large volume of multi-dimensional omics data, as well as the complex network of host-pathogen interactions, the limitations of traditional methods have become increasingly prominent (Chetty and Blekhman, 2024; Amarapathy and Tyagi, 2026). Fortunately, the rapid development of machine learning (ML) and artificial intelligence (AI) is advancing our comprehension of microbial pathogenic mechanisms and drug tolerance to unprecedented levels of both depth and breadth (Wong et al., 2023; Odone et al., 2026).

This Research Topic offers ten insightful studies, highlighting the significance of AI/ML technologies in pathogenic microorganism research, covering core directions such as virulence factors mining, antimicrobial resistance phenotype prediction, and personalized risk stratification.

## Precise prediction and clinical decision support

A series of studies have focused on using AI/ML to establish highly accurate clinical risk prediction models. Jeon et al. employed the XGBoost algorithm to predict the risk of bloodstream infection in carbapenem-resistant *Enterobacteriaceae* carriers within 30-day and 14-day windows, achieving area under the curve (AUC) values of 0.917 and 0.854 respectively. The minimum inhibitory concentration of carbapenems and the neutrophil count were identified as important predictors. Chen et al. selected patients with pyogenic liver abscess as the research subjects and employed feature selection methods such as LASSO regression and Boruta analysis to establish a logistic regression model for predicting multiple organ dysfunction syndrome. The model achieved better discriminative ability than the traditional qSOFA scoring system and identified independent risk factors including invasive *Klebsiella pneumoniae* liver abscess syndrome, septic shock, elevated international normalized ratio, and ascites.

Pediatric patients were recruited as the research subjects, Qin et al. based on a retrospective pediatric cohort, established an XGBoost model to predict carbapenem-resistant *Pseudomonas aeruginosa* infections in children. The internal-validation AUC reached 0.848, and retained satisfactory stable discriminatory ability in the independent temporal validation cohort (AUC = 0.715). The length of hospital stay, CD8+ T cell count, ventilator use, and carbapenem exposure were identified as the top influential predictive factors. Li et al. conducted a multicenter study and developed a random forest model implemented as a web-based application for predicting sepsis in patients with multidrug-resistant *P. aeruginosa* infection. The external-validation AUC reached 0.816, and the serum calcium levels, chronic obstructive pulmonary disease, red blood cell distribution width-standard deviation (RDW-SD), and abdominal infection were found to be key predictive factors.

In the field of intensive care units, Yang et al. utilized public databases to construct and compare eight distinct ML models, and found that the support vector machine performed the best in predicting *Escherichia coli* infection (AUC = 0.745). They identified gender, age, sepsis, and serum potassium level as important predictive factors, and developed a web-based real-time tool for assessing clinical risks. Gao et al. addressed the high-mortality complication caused by carbapenem-resistant *Acinetobacter baumannii* infection in postoperative intensive care unit (ICU) patients. They built predictive models using eight ML algorithms, and found that the gradient boosting algorithm as the optimal in risk prediction, with an AUC of 0.867. They further identified mechanical ventilation duration, central venous catheterization, ICU length of stay, and carbapenem exposure as the four most influential predictors.

## Broadening the scope of research on pathogens and drug discovery

The AL/ML applications go far beyond clinical prediction. Farag et al. utilized AI tools to develop an analytical pipeline for predicting the structures and functions of species-specific secreted proteins of emerging pathogens. From the *African Emomonella* isolates, they identified multiple protein domains including toxins, allergens, adhesins, hydrolases, and inhibitors, and also discovered a lysostaphin-like protein with excellent MHC-II binding ability. Stoia et al. analyzed the key limitations and challenges of current AI-driven strategies for gastrointestinal-kidney infections and proposed an AI-integrated framework combining clinical, microbiological and multi-omics data to enhance treatment efficacy, reduce antibiotic resistance and preserve microbiome integrity.

## Optimizing the diagnostic process and molecular epidemiological surveillance

To accelerate the reporting workflow of clinical microbiological testing, Zhang et al. employed the random forest to develop a non-culture-dependent model for early differentiation between *E. coli* and non-*E. coli* urinary tract infections. They found that gender, lymphocyte counts (LYM), and alanine aminotransferase (ALT) were the most influential predictive factors. Although its test-set discriminatory performance is moderate, the model could still inform early decisions before culture results are available. Fan et al. constructed a molecular transmission network of 334 HIV-1-infected individuals aged ≥ 50 years and found that the overall pre-treatment drug resistance prevalence to be 12.3%. Divorce/widowhood, specific residential areas, and CRF07_BC infection were independent risk factors for inclusion in the HIV transmission cluster.

## Summary and outlook: the future of intelligent anti-infection

These achievements collectively signal a shift in the anti-infection paradigm: from a passive response to a data- and algorithm-driven approach of proactive prediction, targeted prevention, and precise intervention (Dazhu et al., 2025; Michael et al., 2026). However, to transform the initial results into widely recognized clinical applications, the participation of all fields is still necessary (Dong et al., 2025). Firstly, external validation and standardization are of great significance. Currently, most existing models are developed based purely on single-center or retrospective studies. In the future, there is an urgent need to conduct prospective, multi-center external validation studies to evaluate the generalization performance and stability of the models. Meanwhile, it is also important to establish standardized protocols for data collection and model reporting (Collins et al., 2024; Sardar et al., 2026). Secondly, data integration and mechanism research are indispensable components for advancing the field. Future predictive and diagnostic models should be designed to systematically incorporate not only standardized data from routine clinical assessments and laboratory examinations, but also multi-omics datasets spanning genomics, transcriptomics, proteomics, and metabolomics host immune status and microbiome characteristics, etc. In this way, we can shift from correlational predictions to a true understanding of causal mechanisms (Yu et al., 2026; Amarapathy and Tyagi, 2026). Finally, and most importantly, the focus is placed on translating models into clinical application, that is, developing user-friendly and real-time interactive tools that can be seamlessly integrating into the clinical workflows. This requires the joint, coordinated efforts of clinicians, data scientists, hospital administrators, and regulatory agencies (Qiao et al., 2024; Waldock et al., 2025).
